# Editorial: Innovation in developmental psychology, education, sports, and arts: advances in research on individuals and groups, volume II

**DOI:** 10.3389/fpsyg.2025.1581203

**Published:** 2025-03-18

**Authors:** Alexandra Predoiu, Georgeta Pânişoară, Andrzej Piotrowski, Radu Predoiu

**Affiliations:** ^1^Sports and Motor Performance Department, Faculty of Physical Education and Sport, National University of Physical Education and Sports, Bucharest, Romania; ^2^Faculty of Psychology and Educational Sciences, University of Bucharest, Bucharest, Romania; ^3^Institute of Psychology, University of Gdańsk, Gdańsk, Poland; ^4^Teachers' Training Department, Faculty of Physical Education and Sport, National University of Physical Education and Sports, Bucharest, Romania

**Keywords:** growth mindset, life skills in children, teenagers and adults, performance improvement, educational development, innovative methods

This Research Topic (now in its second volume) aims to produce valuable insights for psychologists, educators, and sports science professionals to promote growth and development in children, adolescents, and adults, thereby enhancing their performance levels.

Human development is a complex process influenced by psychological, educational, and physical factors that shape individuals' cognitive, emotional, and social identity and wellbeing. Psychological aspects such as resilience, emotional intelligence, and social support play a crucial role in mental health and behavior regulation, affecting academic performance and personal development. Studies on aggression, bullying, and family resilience highlight the importance of fostering supportive environments to promote emotional stability and prevent negative behavioral patterns. Understanding these factors is essential for creating interventions that enhance mental health and social integration, particularly among children and adolescents.

Education and personal development are equally significant in shaping individuals' cognitive abilities and life skills. Motivation, parenting styles, and early childhood environments influence language development, non-cognitive skills, and academic achievement. Additionally, innovative pedagogical methods and assessment tools contribute to optimizing learning processes, ensuring that students receive comprehensive support for intellectual and social growth.

Physical health and sports further complement psychological and educational development by enhancing motor skills and emotional resilience. Regular physical activity and sports programs have been shown to improve mental health, social relationships, and stress management.

The manuscripts in this Research Topic explore interdependent themes, which we will group and suggestively name below.

The first theme, *Emotional and Behavioral Development Factors*, focuses on psychological factors influencing individual behavior, including aggression, emotional resilience, mental health, and interpersonal relationships. The authors investigated aggressive behaviors in different contexts: school vs. sports. The study by Rus et al. underlines the significant link between conflicts in the school environment and aggressive behaviors, while Patenteu et al. draw attention to the role of foul (violent) play in athletes' risky behaviors. Moreover, articles have investigated how resilience, emotional intelligence, and family support contribute to preventing negative behaviors and enhancing mental wellbeing. Children's emotional states, parental coping strategies, and family resilience were examined by Vladislav et al. The authors discussed the importance of adaptive coping strategies within the family system during a period of crisis, with maladaptive strategies being linked to anxiety, depression, and stress. In the context of a possible period of crisis, Yang et al. stressed that the crucial role of physical activity in generating positive emotions, interpersonal forgiveness, and emotional intelligence is also positively influenced. In a sports setting (runners being investigated), it seems that not only athletes but also their partners need to be physically active in a crisis situation (such as the COVID-19 pandemic). For the quality of interpersonal relationships/marital satisfaction, the research by Vilaregut et al. increased awareness of the role of exercise on mental health. Specific concepts of positive psychology, such as happiness, resilience, hope, gratitude, character, mindfulness, and growth mindsets, were examined by Platt et al. Integrating these concepts into daily life improves symptoms of mental distress and levels of wellbeing.

The second theme we identified in our Research Topic is *Education and Personal Development*. This theme addresses motivation, learning, cognitive and motor development of children and students, and factors influencing academic performance. Regarding motivation and educational performance, Li et al. emphasized the essential role of students' mentorship homegate (or team) support, which encourages creative behaviors, with intrinsic motivation being a mediator. The authors highlighted the need for higher education institutions to cultivate a challenging research environment that positively impacts students' intrinsic motivation. In the context of educational performance, research by Peng and Zhang revealed that achievement motivation has a positive and significant impact on the educational practice skills of pre-service teachers. Researchers underlined that achievement motivation is an internal drive to pursue and accomplish objectives (Wu et al., 2017), influencing work responsibility and learning engagement. Considering the cognitive development in children, Qiu and Wang, and Wang and Zheng examined how the home environment and parenting styles influence non-cognitive development and language skills. In the first case, it was found that the home environment predicts the language development of toddlers, with executive function playing a mediating role, while children's temperamental characteristics should also be taken into account. In terms of parenting style, the authoritative and authoritarian parenting styles were investigated. Wang and Zheng emphasized the positive impact of the authoritative style and the negative impact of the authoritarian style on the non-cognitive abilities of students (e.g., interpersonal skills, resilience), with a greater impact being observed in girls. It is worth mentioning that the family's socioeconomic status influences the parenting style and represents an important indicator of the home environment (Chow et al., 2017), the most influential microsystem on childhood development. Not least, children's motor skill development, linked to pedagogical approaches and assessment tools, was investigated. The study by Ghorbanzadeh et al. recommends the combined method of sports education and teaching games for understanding to improve children's motor proficiency. However, the perceived motor competencies (PMCs) are also important, along with the actual motor competencies, in children's motor and psychological development. Bretz et al. proposed the SEMOK-1-2 instrument, which can be economically used for assessing children's perceived motor competencies and is suitable for monitoring PMCs during physical education.

Another subject can be identified as theme no. 3—*Health and Sports*. This theme examines the influence of psychological phenomena on emotional health and performance. Predoiu et al. highlighted the importance of investigating athletes' subconscious levels to increase the likelihood of sports performances. Based on the dual processing model (Gawronski and Bodenhausen, 2006), authors explored the implicit/indirect aggression of athletes using an implicit association test (IAT), being aware that the automated/unconscious way of processing information contributes to and defines human behavior (Richetin and Richardson, 2008). The research by Zhou et al. underlines the existence of causal relationships between physical activity, social support, and family relationships in college students. Precisely, family relations represent a causal variable for physical activity behaviors, while social support represents a causal variable for both family relationships and physical activity behaviors. We also found research, like that conducted by Hong and Minikin, which offers insights into practitioners' perceptions and experiences of the *Voices of Athletes*—a specialized athlete support program that plays a critical role in empowering athletes, preparing them for post-sport life, and helping them become leaders within their communities.

Research in this Research Topic provides valuable insights into how educational practices can be improved to foster lifelong learning and adaptability in an evolving world. Studies on aggression in sports, the impact of the COVID-19 pandemic on athletes, and the role of exercise in emotional regulation emphasize the interconnectedness of physical and psychological wellbeing. By integrating these perspectives, educators and researchers can develop holistic approaches that support healthy development across multiple life domains. The important insights that the Research Topic generates assist professionals, such as teachers, social workers, psychologists, and coaches, in supporting children, adolescents, or adults, encouraging them to consistently focus on their capacity to learn and evolve.
